# ﻿Two new species of *Sinolachnus* Hille Ris Lambers (Hemiptera, Aphididae, Lachninae) from China

**DOI:** 10.3897/zookeys.1182.110322

**Published:** 2023-10-12

**Authors:** Zhao-Xu Li, Jing Chen, Li-Yun Jiang, Ge-Xia Qiao

**Affiliations:** 1 Key Laboratory of Zoological Systematics and Evolution, Institute of Zoology, Chinese Academy of Sciences, No. 1-5 Beichen West Road, Chaoyang District, Beijing 100101, China Key Laboratory of Zoological Systematics and Evolution, Institute of Zoology, Chinese Academy of Sciences Beijing China; 2 College of Life Science, University of Chinese Academy of Sciences, No. 19, Yuquan Road, Shijingshan District, Beijing 100049, China University of Chinese Academy of Sciences Beijing China

**Keywords:** Alate, aphid, apterous, key, morphology, taxonomy

## Abstract

Two new *Sinolachnus* species from China, *Sinolachnusrubusis* Qiao & Li, **sp. nov.** feeding on *Rubus* sp. from Shaanxi and Sichuan Provinces, and *Sinolachnusyunnanensis* Qiao & Li, **sp. nov.** feeding on *Elaeagnus* sp. from Yunnan Province, are described and illustrated. Keys to *Sinolachnus* species distributed in China are presented. All examined specimens are deposited in the National Zoological Museum of China, Institute of Zoology, Chinese Academy of Sciences, Beijing, China.

## ﻿Introduction

The aphid genus *Sinolachnus* was established by Hille Ris Lambers (1956), with *Lachnusniitakayamensis* Takahashi as type species. Subsequently, Tao (1989) described a second species, *Sinolachnustaiwanus* Tao, only based on alatae collected by a malaise trap. Chakrabarti and Das (2015) reported a new species, *Sinolachnuselaeagnensis* Chakrabarti & Das from Bhutan, with descriptions of alate viviparous females and fourth instar nymphs. Kanturski et al. (2022 [2023]) revised the genus, proposed three new species, *Sinolachnusnipponicus* Kanturski, Yeh & Lee, *Sinolachnustakahashii* Kanturski, Yeh & Lee and *Sinolachnusyushanensis* Kanturski, Yeh & Lee, suggested two new combinations, *Sinolachnusplurisensoriatus* (Zhang) (from *Cinara* Curtis) and *Sinolachnusrubi* (Ghosh & Raychaudhuri) (from *Maculolachnus* Gaumont), and transferred the genus from Tuberolachnini Oestlund to Tramini Herrich-Schaeffer. So far, eight species are recorded in *Sinolachnus*, including five species distributed in China (Kanturski et al. 2022 [2023]).

*Sinolachnus* is distinguished within Lachninae by the presence of numerous protuberant secondary rhinaria on the antennae of alatae (Tao 1961; Ghosh 1982; Blackman and Eastop 1994), which was further demonstrated as a reliable characteristic of the genus (Kanturski et al. 2022 [2023]); other diagnostic generic characteristics were provided including the arrangement of accessory rhinaria on antennal segment VI and several “sense pegs” on the first tarsal segments.

Herein, two new species, *Sinolachnusrubusis* Qiao & Li, sp. nov. feeding on *Rubus* sp. (Rosaceae) from Shaanxi and Sichuan Provinces, and *Sinolachnusyunnanensis* Qiao & Li, sp. nov. feeding on *Elaeagnus* sp. (Elaeagnaceae) from Yunnan Province, China, are described and illustrated. Keys to apterae and alatae of *Sinolachnus* species distributed in China are provided.

## ﻿Material and methods

### ﻿Morphological description

Aphid terminology and the measurements in this paper generally follow Blackman and Eastop (1994) and Kanturski et al. (2022 [2023]). The unit of measurement is millimeter (mm). The following abbreviations are used:

**Ant. I, II, III, IV, V, VIb** antennal segment I, II, III, IV, V and the base of segment VI, respectively;

**PT** processus terminalis;

**Ant. III BD** basal diameter of antennal segment III;

**URS** ultimate rostral segment;

**BW URS** basal width of ultimate rostral segment;

**MW hind tibia** mid-width of hind tibia;

**HT Ib** basal width of first hind tarsal segment;

**HT Id** dorsal length of first hind tarsal segment;

**HT Iv** ventral length of first hind tarsal segment;

**HT II** second hind tarsal segment;

**BW SIPH** basal width of siphunculus;

**DW SIPH** distal width of siphunculus;

**BW Cauda** basal width of cauda;

**Frontal setae** the longest seta on vertex;

**Setae on Ant. III** the longest seta on antennal segment III;

**Setae on Hind tibia** the longest seta on hind tibia;

**Setae on Tergite I** the longest marginal seta on abdominal tergite I;

**Setae on Tergite VIII** the longest seta on abdominal tergite VIII.

### ﻿Specimen depositories

The holotype and paratypes of the new species are deposited in the National Zoological Museum of China, Institute of Zoology, Chinese Academy of Sciences, Beijing, China.

## ﻿Taxonomy

### ﻿ Sinolachnus rubusis

Taxon classificationAnimaliaHemipteraAphididae

Qiao & Li sp. nov.

B2ABF7E0-F0D0-591C-892B-05A744250F31

https://zoobank.org/E2426EA3-0D93-4EA5-AFD9-9A5F9A1DD78F

Figs 1–15 , 16–27 , 28–33 , 34–35 , Table 1

#### Type material.

***Holotype***: apterous viviparous female, CHINA: Shaanxi Province (Baoji City, Tongtianhe National Forest Park, 34.2133°N, 106.5861°E, altitude 1650 m), 12 July 2016, No. 37534-1-1, on *Rubus* sp., coll. R. Chen and C.C. Du. ***Paratypes***: seven apterous viviparous females and one alate viviparous female, with the same collection data as holotype; one apterous viviparous female and three alate viviparous females, CHINA: Sichuan Province (Ya’an City, Zhougong Mountain), 14 July 2018, No. 43462, on *Rubus* sp., coll. Yong Wang.

#### Etymology.

The new species is named after the genus name of its host plant, *rubusis* being the masculine form.

#### Diagnosis.

In apterae, abdominal tergites IV–VII with scattered sclerites, pleural and marginal sclerites often incompletely fused (Fig. 16). In alatae, antennae with fewer round and protuberant secondary rhinaria in various sizes, Ant. III–VI with 29–54, 5–18, 3–14, 4–8 secondary rhinaria, respectively; abdominal tergite VII without sclerites.

#### Description.

**Apterous viviparous female**: Body oval, reddish-brown in life, dorsal patches and siphunculi dark brown (Fig. 34).

***Mounted specimens.*** Head, antennal segments except basal half of Ant. III, rostral segments III–V, pronotum, mesonotum, distal half of tibiae, tarsi, siphunculi, cauda, anal plate and genital plate dark brown; other parts pale brown; coxae, trochanters, femora and basal half of tibiae pale yellowish-brown; setae on metanotum and abdominal tergites bearing dark base-sclerites. For morphometric data, see Table 1.

**Table 1.** Morphometric data of *Sinolachnusrubusis* Qiao & Li, sp. nov. (measurements in mm, with means in brackets).

	Characters	Apterous viviparous females (*N* = 9)	Alate viviparous females (*N* = 4)
Length (mm)	Body length	3.19–3.67 (3.42)	3.65–3.69 (3.67)
	Body width	1.97–2.47 (2.25)	1.73–1.85 (1.79)
	Antenna	1.56–1.84 (1.71)	1.66–1.88 (1.73)
	Ant. I	0.14–0.17 (0.15)	0.14–0.15 (0.14)
	Ant. II	0.11–0.13 (0.12)	0.10–0.11 (0.10)
	Ant. III	0.59–0.77 (0.69)	0.69–0.79 (0.72)
	Ant. IV	0.20–0.26 (0.23)	0.21–0.28 (0.24)
	Ant. V	0.21–0.26 (0.22)	0.23–0.26 (0.24)
	Ant. VIb	0.16–0.22 (0.19)	0.18–0.20 (0.19)
	PT	0.09–0.11 (0.10)	0.09–0.10 (0.09)
	URS	0.24–0.26 (0.25)	0.23–0.25 (0.24)
	Hind femur	1.15–1.34 (1.27)	1.35–1.44 (1.40)
	Hind tibia	1.90–2.28 (2.13)	2.28–2.51 (2.39)
	HT Ib	0.05–0.06 (0.05)	0.05
	HT Id	0.02	0.02
	HT Iv	0.10–0.12 (0.11)	0.10–0.11 (0.10)
	HT II	0.31–0.37 (0.33)	0.31–0.35 (0.33)
	BW SIPH	0.36–0.43 (0.40)	0.41–0.44 (0.42)
	DW SIPH	0.11–0.12 (0.11)	0.10
	Cauda	0.13–0.15 (0.14)	0.12–0.13 (0.12)
	BW Cauda	0.31–0.40 (0.36)	0.26–0.32 (0.28)
	Ant. III BD	0.04–0.05 (0.05)	0.04–0.05 (0.04)
	MW hind tibia	0.09–0.10 (0.09)	0.08–0.09 (0.08)
	Frontal setae	0.09–0.12 (0.10)	0.10–0.11 (0.11)
	Setae on Tergite I	0.09–0.11 (0.10)	0.10–0.13 (0.11)
	Setae on Tergite VIII	0.11–0.14 (0.13)	0.11–0.13 (0.12)
	Setae on Ant. III	0.08–0.10 (0.09)	0.10–0.11 (0.10)
	Setae on Hind tibia	0.09–0.10 (0.09)	0.11–0.12 (0.11)
Ratio (times)	Body length/Body width	1.40–1.62 (1.52)	1.99–2.11 (2.05)
	Whole antenna/Body	0.44–0.57 (0.50)	0.45–0.51 (0.48)
	Hind femur/Ant. III	1.74–1.93 (1.85)	1.82–1.94 (1.88)
	Hind tibia/Body	0.56–0.69 (0.62)	0.62–0.68 (0.65)
	PT/Ant. VIb	0.41–0.62 (0.51)	0.44–0.54 (0.49)
	URS/BW URS	2.63–3.37 (2.90)	2.93–3.21 (3.10)
	URS/HT II	0.65–0.81 (0.75)	0.71–0.77 (0.75)
	HT Ib/HT Id	2.26–3.47 (2.68)	2.25–2.71 (2.44)
	HT Ib/HT Iv	0.42–0.48 (0.46)	0.41–0.48 (0.44)
	Frontal setae/Ant. III BD	1.87–2.86 (2.18)	2.28–2.80 (2.56)
	Setae on Tergite I/Ant. III BD	1.89–2.43 (2.16)	2.32–3.18 (2.64)
	Setae on Tergite VIII/Ant. III BD	2.49–3.09 (2.78)	2.66–3.05 (2.88)
	Setae on Ant. III/Ant. III BD	1.74–2.38 (1.96)	2.21–2.65 (2.42)
	Setae on Hind tibia/MW hind tibia	0.95–1.09 (1.01)	1.30–1.57 (1.43)
	DW SIPH/BW SIPH	0.26–0.32 (0.29)	0.23–0.24 (0.23)
	Cauda/BW Cauda	0.38–0.43 (0.41)	0.40–0.50 (0.44)

**Figures 1–15.**
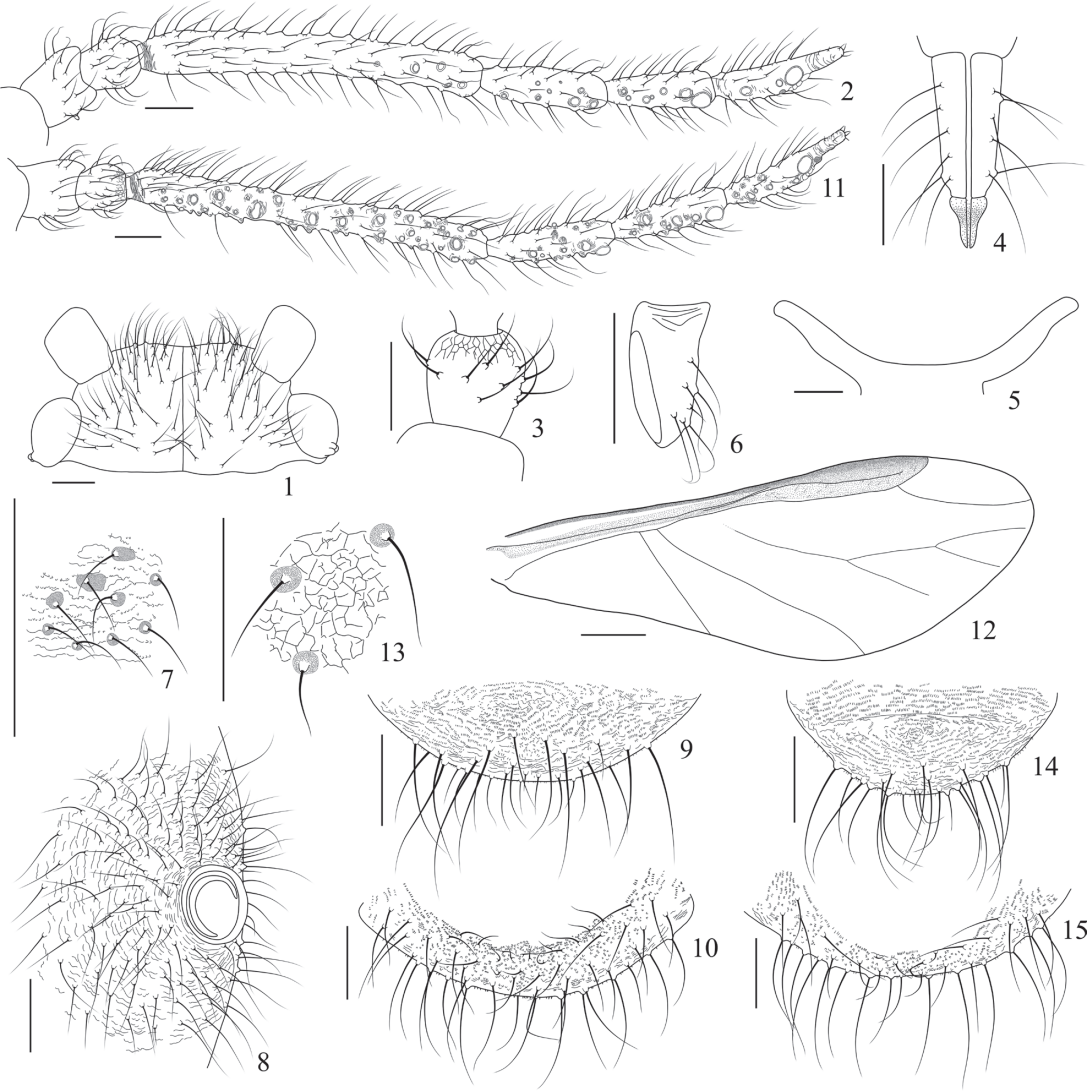
*Sinolachnusrubusis* Qiao & Li, sp. nov. Apterous viviparous female: **1** dorsal view of head **2** antenna **3**Ant. II, distal reticulations and setae shown **4**URS**5** mesosternal furca **6** hind first tarsal segment **7** spinulose imbrications and setae on abdominal tergites **8** siphunculus **9** cauda **10** anal plate. Alate viviparous female: **11** antenna **12** fore wing **13** reticulations and setae on abdominal tergites **14** cauda **15** anal plate. Scale bars: 0.10 mm (**1–11, 13–15**); 0.50 mm (**12**). (All figures were drawn according to type material No. 37534).

***Head.*** Head dorsum smooth, with an obvious dark median suture. Head with 88–111 long and pointed dorsal setae. Frons round. Ocular tubercles well developed (Figs 1, 18). Antennae almost smooth (Figs 2, 19), distal part of Ant. II with polygonal reticulations on dorsal (Fig. 3), basal part of Ant. III and PT with transverse striae. Antennal setae fine, long and pointed, Ant. I–VI each with 20–28, 18–30, 94–139, 30–40, 34–40, 25–35+2–3 setae, respectively; apex of PT with 3–5 short blunt setae. Primary rhinaria elliptical, Ant. VI with 3–6 accessory rhinaria around primary rhinaria; secondary rhinaria almost round and protuberant in various sizes, basal diameter of secondary rhinaria about 0.006–0.038 mm, Ant. III–VI with 1–8, 2–11, 0–6 and 0–7 secondary rhinaria, respectively, along the distal part of Ant. III, the middle and distal part of Ant. IV, the entire length of Ant. V and Ant. VIb, respectively. Rostrum long, beyond hind coxae; URS wedge-shaped (Figs 4, 20), with 2–3 pairs of primary setae and 10–14 accessory setae.

**Figures 16–27.**
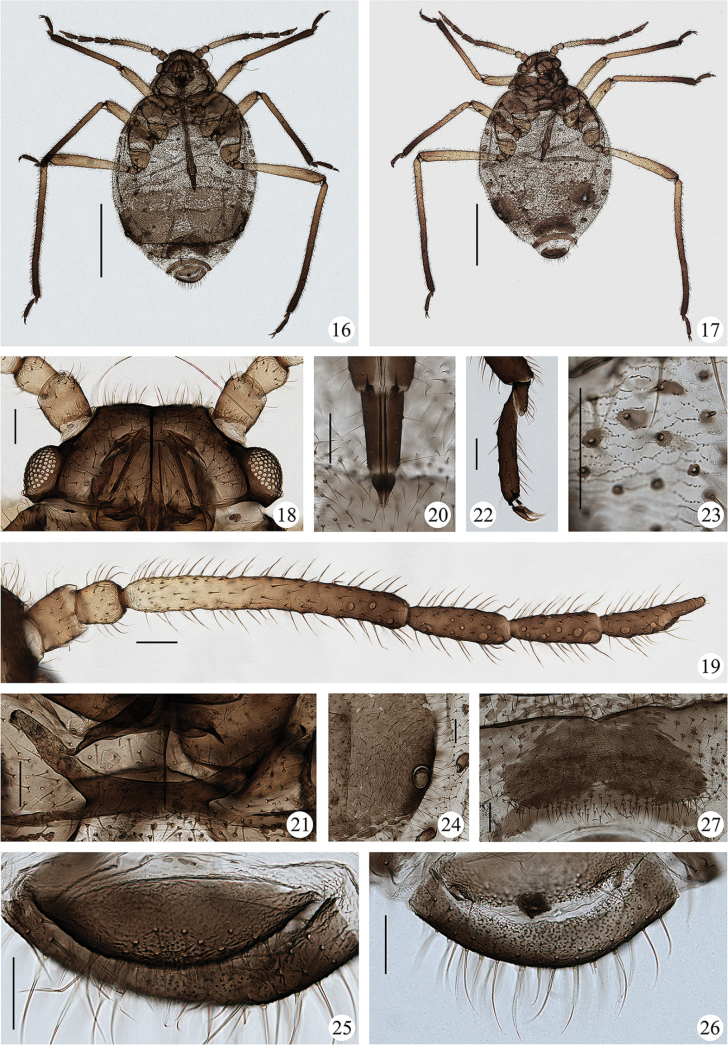
*Sinolachnusrubusis* Qiao & Li, sp. nov. Apterous viviparous female: **16** dorsal view of body with large fused sclerites **17** dorsal view of body with scattered sclerites **18** dorsal view of head **19** antenna **20**URS**21** mesosternal furca **22** hind tarsi and claws **23** spinulose imbrications on abdominal tergites **24** siphunculus **25** cauda **26** anal plate **27** genital plate. Scale bars: 1.00 mm (**16, 17**); 0.10 mm (**18–27**). (All figures were photographed according to type material No. 37534)

***Thorax.*** Pronotum and mesonotum with a few scattered spinules; metanotum with spinulose imbrications and small scattered sclerites, pleural and marginal sclerites sometimes fused. Dorsal setae numerous, fine and pointed. Mesosternal furca with a short stem (Figs 5, 21). Legs normal, with long and pointed setae. First tarsal chaetotaxy: 12–16, 9–14, 8–10; first fore tarsal segments with 6–9 peg-like setae and 5–8 long setae, first mid-tarsal segments with 3–6 peg-like setae and 5–9 long setae, first hind tarsal segments with 1–3 peg-like setae and 6–9 long setae.

***Abdomen.*** Abdominal tergites I–VI with spinulose imbrications (Figs 7, 23), tergites VII, VIII and venter with spinulose stripes. Abdominal tergites I–III with a few small scattered sclerites, sclerites on tergite III more obvious than tergites I and II; tergites IV–VII with scattered sclerites, pleural and marginal sclerites often incompletely fused (Fig. 16), sometimes sclerites reduced (Fig. 17); tergite VIII with a transverse band; intersegmental muscle sclerites small and dark. Dorsal setae numerous, long and pointed. Abdominal tergite VIII with 31–54 setae. Spiracles round to oval, open or closed, on brown spiracular plates. Siphunculi truncate, on dark brown seta-bearing cones, with flange and transverse striae (Figs 8, 24), surrounding by 88–142 setae. Cauda round with spinulose stripes, with 28–36 long or short setae (Figs 9, 25). Anal plate broadly round with spinules, with 66–88 long or short setae (Figs 10, 26). Genital plate transverse elliptical with spinulose stripes, with 100–129 setae (Fig. 27). Genopophyses three, each with 10–14, 9–12, 8–13 setae, respectively.

**Alate viviparous female**: Body elongate-oval, brown in life, with dark brown siphunculi (Fig. 35).

***Mounted specimens.*** Head, antennae, rostral segments III–V, thorax, legs except basal half of tibiae, siphunculi, cauda, anal plate and genital plate dark brown, other parts pale brown; dorsal setae on abdominal tergites bearing dark base-sclerites. For morphometric data, see Table 1.

***Head.*** Head dorsum smooth with an obvious dark median suture. Head with 70–88 long and pointed dorsal setae. Frons flat. Ocular tubercles well developed. Antennae almost smooth (Figs 11, 29), distal part of Ant. II with polygonal reticulations and distinct on dorsal, obvious or weak on ventral; basal part of Ant. III and PT with transverse striae. Antennal setae long and pointed, Ant. I–VI each with 22–24, 18–29, 88–129, 21–38, 33–43, 24–32+2–3 setae, respectively; apex of PT with 4–6 short blunt setae. Primary rhinaria elliptical, Ant. VI with 5 accessory rhinaria around primary rhinaria; secondary rhinaria almost round and protuberant in various sizes, basal diameter of secondary rhinaria about 0.008–0.046 mm, Ant. III–VI with 29–54, 5–18, 3–14, 4–8 secondary rhinaria respectively along the entire length of Ant. III–V and base of Ant. VI. Rostrum long, reaching hind coxae; URS wedge-shaped, with 3 pairs of primary setae and 9–12 accessory setae.

**Figures 28–33.**
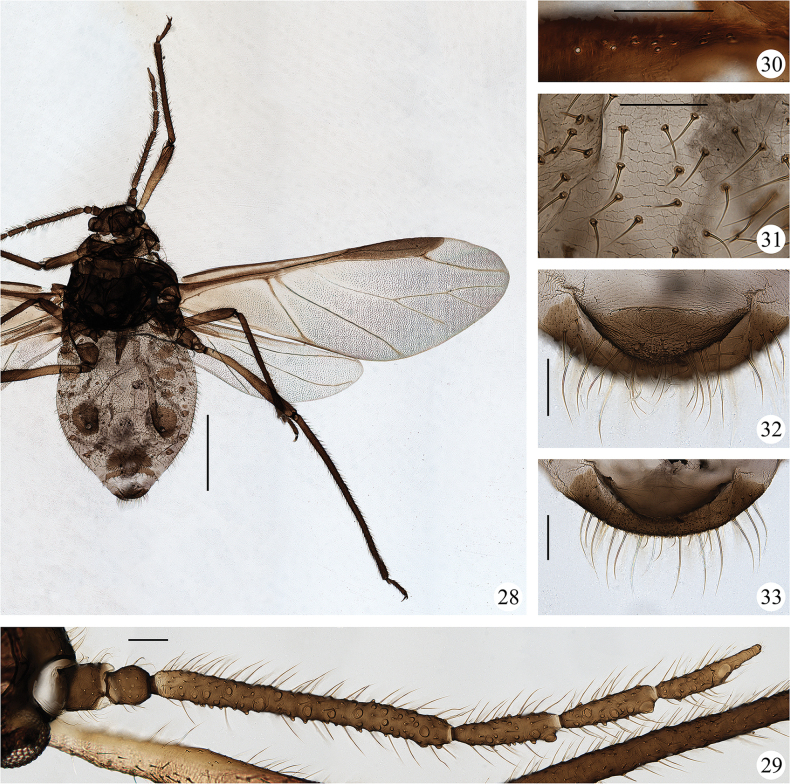
*Sinolachnusrubusis* Qiao & Li, sp. nov. Alate viviparous female: **28** dorsal view of body **29** antenna **30** sensilla on subcostal of fore wing **31** reticulations on abdominal tergites **32** cauda **33** anal plate. Scale bars: 1.00 mm (**28**); 0.10 mm (**29–33**). (All figures were photographed according to type material No. 37534)

***Thorax.*** Legs normal, with long and pointed setae. First tarsal chaetotaxy: 10–15, 10, 6–9; first fore tarsal segments with 6–11 peg-like setae and 4 or 5 long setae, first mid-tarsal segments with 4 or 5 peg-like setae and 5 or 6 long setae, first hind tarsal segments with 2 peg-like setae and 4–7 long setae. Wings with scaly imbrications entirely (Fig. 28); campaniform sensilla near the base of subcosta slightly protuberant (Fig. 30), fore wings and hind wings with 10–13 and 11–20 campaniform sensilla on basal part, respectively; fore wings with pale media twice branched and faint on basal part (Fig. 12), pterostigma with 25–27 setae; hind wings with two oblique veins.

***Abdomen.*** Abdominal tergites smooth, tergites I–VI with polygonal reticulations (Figs 13, 31), tergites VII, VIII and venter with spinulose stripes. Abdominal tergites II–IV each with 1 pair of marginal sclerites, sclerites on tergite IV relatively smaller, tergite VIII with a transverse band; intersegmental muscle sclerites small and dark. Dorsal setae on abdomen long and pointed, relatively sparse than on venter. Abdominal tergite VIII with 29–33 setae. Spiracles round and closed, on brown spiracular plates. Siphunculi truncate, on dark brown seta-bearing cones, with flange and transverse striae, surrounding by 123–156 setae. Cauda elliptical with spinulose stripes, with 26–30 long or short setae (Figs 14, 32). Anal plate broadly round with spinules, with 54–64 setae (Figs 15, 33). Genital plate transverse elliptical with spinulose stripes, with 87–105 setae. Genopophyses three, each with 12, 14, 12 setae.

#### Distribution.

China (Shaanxi, Sichuan).

#### Host plant.

*Rubus* sp. (Rosaceae).

#### Biology.

The species feeds on roots of host plants and was visited by ants.

#### Comments.

Apterae of the new species are related to *Sinolachnusrubi* in having abdominal tergites with scattered sclerites and sometimes fused. *Sinolachnusrubi* was originally regarded as a member of *Maculolachnus*, but transferred to *Sinolachnus* by Kanturski et al. (2022 [2023]). Based on the detailed description, the new species obviously differs from *S.rubi* as follows: femora and basal half of tibiae pale yellowish-brown, distal half of tibiae dark brown (the latter: basal half of femora slightly pale, distal half of femora and tibiae dark brown); HT Ib 2.26–3.47 times as long as HT Id (the latter: 1.80–2.00 times); HT II 0.31–0.37 mm in length, URS 0.65–0.81 times as long as HT II (the latter: HT II 0.23–0.28 mm in length, URS 0.83–0.96 times as long as HT II); abdominal tergites I–III with a few small scattered sclerites, tergites IV–VII with scattered sclerites, pleural and marginal sclerites often incompletely fused (the latter: abdominal tergites with many small scattered sclerites, often fused in spinal parts, form bands on tergites I and VII), tergite VIII with 31–54 setae (the latter: 18–20 setae). In addition, two mentioned species specially infest *Rubus* sp., the new species feeds on roots of host plants, while *S.rubi* was recorded from apical stems.

### ﻿ Sinolachnus yunnanensis

Taxon classificationAnimaliaHemipteraAphididae

Qiao & Li sp. nov.

D960AC45-D9AE-5EBC-82D5-34DF680BC0D3

https://zoobank.org/2CA4D6AA-7B90-4CCF-9D72-0A608A74904D

Figs 36–45 , 46–58 , 59–64 , Table 2

#### Type material.

***Holotype***: apterous viviparous female, CHINA: Yunnan Province (Nujiang Lisu Autonomous Prefecture, 26.4401°N, 99.3911°E, alt. 2341 m), 28 July 2022, No. 54113-1-1, on *Elaeagnus* sp., coll. S. Xu and Ying Wang; ***Paratypes***: one alate viviparous female, others same as holotype; two apterous viviparous females and one alate viviparous female, CHINA: Yunnan Province (Nujiang Lisu Autonomous Prefecture, 26.5618°N, 99.4392°E, alt. 2774 m), 1 August 2022, No. 54211, on *Elaeagnus* sp., coll. S. Xu and Ying Wang; two apterous viviparous females, CHINA: Yunnan Province (Diqing Tibetan Autonomous Prefecture, 27.3449°N, 99.2376°E, alt. 2529 m), 2 August 2022, No. 54223-1-1, on *Elaeagnus* sp., coll. S. Xu and Ying Wang; two apterous viviparous females, CHINA: Yunnan Province (Diqing Tibetan Autonomous Prefecture, 27.3407°N, 99.2448°E, alt. 2558 m), 2 August 2022, No. 54230-1-1, on *Elaeagnus* sp., coll. S. Xu and Ying Wang; two apterous viviparous females, CHINA: Yunnan Province (Diqing Tibetan Autonomous Prefecture, 27.2209°N, 99.2755°E, alt. 2152 m), 3 August 2022, No. 54252-1-1, on *Elaeagnus* sp., coll. S. Xu and Ying Wang; one apterous viviparous female and one apterous nymph, CHINA: Yunnan Province (Diqing Tibetan Autonomous Prefecture, 27.1958°N, 99.3338°E, alt. 2395 m), 4 August 2022, No. 54260-1-1, on *Elaeagnus* sp., coll. S. Xu and Ying Wang; two apterous viviparous females, CHINA: Yunnan Province (Diqing Tibetan Autonomous Prefecture, 27.1965°N, 99.3306°E, alt. 2347 m), 4 August 2022, No. 54271-1-1, on *Elaeagnus* sp., coll. S. Xu and Ying Wang; two apterous viviparous females, CHINA: Yunnan Province (Diqing Tibetan Autonomous Prefecture, 27.1976°N, 99.3210°E, alt. 2289 m), 4 August 2022, No. 54272-1-1, on *Elaeagnus* sp., coll. S. Xu and Ying Wang; two apterous viviparous females, CHINA: Yunnan Province (Diqing Tibetan Autonomous Prefecture, 27.2708°N, 99.2311°E, alt. 2215 m), 4 August 2022, No. 54279-1-1, on *Elaeagnus* sp., coll. S. Xu and Ying Wang; CHINA: Yunnan Province (Lijiang City, 26.7731°N, 100.0227°E, alt. 2880 m), 12 August 2022, No. 54424-1-1, on *Elaeagnus* sp., coll. S. Xu and Ying Wang.

#### Etymology.

The new species is named after its distribution location, *yunnanensis* being the masculine form.

#### Diagnosis.

Body relatively small, less than 3 mm in length. PT with 2–6 long setae on basal part. Abdominal tergites of apterous viviparous females often with small scattered spinal sclerites and sometimes fused or unobvious. Alate viviparous females with fewer secondary rhinaria, Ant. III–VI with 70–80, 14, 8, 3 secondary rhinaria, respectively; fore wings with media once branched; abdominal tergite VII with a broad transverse patch with irregular margin.

#### Description.

**Apterous viviparous female**: Body oval, with densely long setae, reddish-brown in life (Figs 61–63), apical or whole antennae and legs, siphunculi, and a transverse patch on abdominal tergite VII dark brown.

***Mounted specimens.*** Head, antennae, rostral segments III–V, pronotum, mesonotum, legs, siphunculi, cauda, anal plate and genital plate dark brown; other parts pale brown; setae on metanotum and abdominal tergites, and some on venter of abdomen bearing dark base-sclerites. For morphometric data, see Table 2.

**Table 2.** Morphometric data of *Sinolachnusyunnanensis* Qiao & Li, sp. nov. (measurements in mm, with means in brackets).

	Characters	Apterous viviparous females (*N* = 10)	Alate viviparous females (*N* = 2)
Length (mm)	Body length	1.94–2.64 (2.34)	2.45–2.49 (2.47)
	Body width	1.15–1.68 (1.48)	0.99–1.27 (1.13)
	Antenna	0.93–1.39 (1.17)	1.38
	Ant. I	0.09–0.12 (0.11)	0.10–0.11 (0.10)
	Ant. II	0.08–0.11 (0.10)	0.10
	Ant. III	0.33–0.52 (0.41)	0.55–0.60 (0.58)
	Ant. IV	0.11–0.19 (0.15)	0.18
	Ant. V	0.12–0.20 (0.16)	0.19
	Ant. VIb	0.13–0.17 (0.15)	0.16
	PT	0.07–0.10 (0.09)	0.09
	URS	0.20–0.25 (0.23)	0.22–0.23 (0.22)
	Hind femur	0.58–0.96 (0.78)	0.98–0.99 (0.99)
	Hind tibia	1.00–1.73 (1.34)	1.76–1.78 (1.77)
	HT Ib	0.03–0.04 (0.04)	0.03
	HT Id	0.01–0.02 (0.01)	0.01–0.02 (0.01)
	HT Iv	0.06–0.09 (0.08)	0.06–0.07 (0.07)
	HT II	0.18–0.24 (0.21)	0.24
	BW SIPH	0.28–0.39 (0.35)	0.28–0.33 (0.30)
	DW SIPH	0.08–0.09 (0.08)	0.07
	Cauda	0.08–0.10 (0.09)	0.09–0.10 (0.09)
	BW Cauda	0.25–0.32 (0.29)	0.23–0.26 (0.24)
	Ant. III BD	0.02–0.03 (0.03)	0.03
	MW hind tibia	0.05–0.07 (0.06)	0.05
	Frontal setae	0.09–0.12 (0.10)	0.10–0.11 (0.10)
	Setae on Tergite I	0.09–0.11 (0.10)	/
	Setae on Tergite VIII	0.09–0.13 (0.11)	0.10–0.12 (0.11)
	Setae on Ant. III	0.10–0.12 (0.10)	0.10–0.11 (0.11)
	Setae on Hind tibia	0.09–0.13 (0.11)	0.10–0.12 (0.11)
Ratio (times)	Body length/Body width	1.47–1.69 (1.59)	1.96–2.47 (2.22)
	Whole antenna/Body	0.45–0.55 (0.50)	0.56
	Hind femur/Ant. III	1.68–2.01 (1.88)	1.66–1.78 (1.72)
	Hind tibia/Body	0.48–0.67 (0.57)	0.72
	PT/Ant. VIb	0.51–0.71 (0.59)	0.57
	URS/BW URS	3.00–3.72 (3.39)	2.93–3.55 (3.24)
	URS/HT II	1.03–1.15 (1.11)	0.92
	HT Ib/HT Id	2.40–3.60 (2.93)	2.07–2.82 (2.44)
	HT Ib/HT Iv	0.43–0.56 (0.49)	0.47–0.48 (0.48)
	Frontal setae/Ant. III BD	3.29–4.83 (3.98)	3.75
	Setae on Tergite I/Ant. III BD	3.07–4.58 (3.82)	/
	Setae on Tergite VIII/Ant. III BD	3.61–5.33 (4.24)	4.14
	Setae on Ant. III/Ant. III BD	3.19–4.86 (4.07)	3.93
	Setae on Hind tibia/MW hind tibia	1.48–2.14 (1.77)	1.91–2.30 (2.10)
	DW SIPH/BW SIPH	0.20–0.28 (0.23)	0.25
	Cauda/BW Cauda	0.28–0.32 (0.30)	0.37–0.40 (0.39)

***Head.*** Head dorsum smooth, with an obvious dark median suture. Head with 104–137 long and pointed dorsal setae. Frons round. Ocular tubercles well developed (Figs 36, 47). Antennae almost smooth (Figs 37, 48), basal part of Ant. III and PT with transverse striae. Antennal setae fine, long and pointed, Ant. I–VI each with 22–30, 28–35, 106–131, 27–38, 29–40, 28–36+3–8 setae, respectively; PT with 5 short blunt setae at apex. Primary rhinaria round, Ant. VI with 4 or 5 accessory rhinaria around primary rhinaria; secondary rhinaria often absent, Ant. III and IV with 1 or 2, Ant. VI with 1 round and protuberant secondary rhinarium occasionally. Rostrum long, reach abdominal segment V; URS elongate wedge-shaped (Figs 38, 49), with 3 pairs of primary setae and 18–26 accessory setae.

**Figures 34–35.**
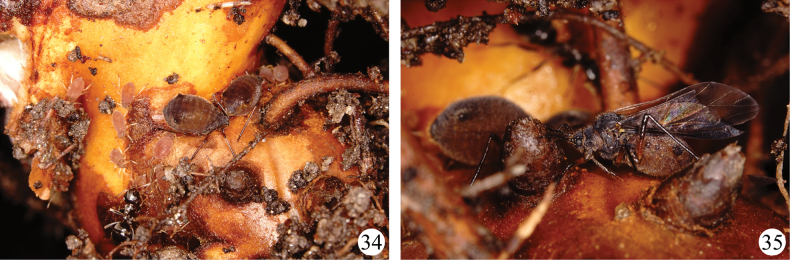
*Sinolachnusrubusis* Qiao & Li, sp. nov. **34** a colony on the root of the host, visited by ants **35** alate viviparous female.

**Figures 36–45.**
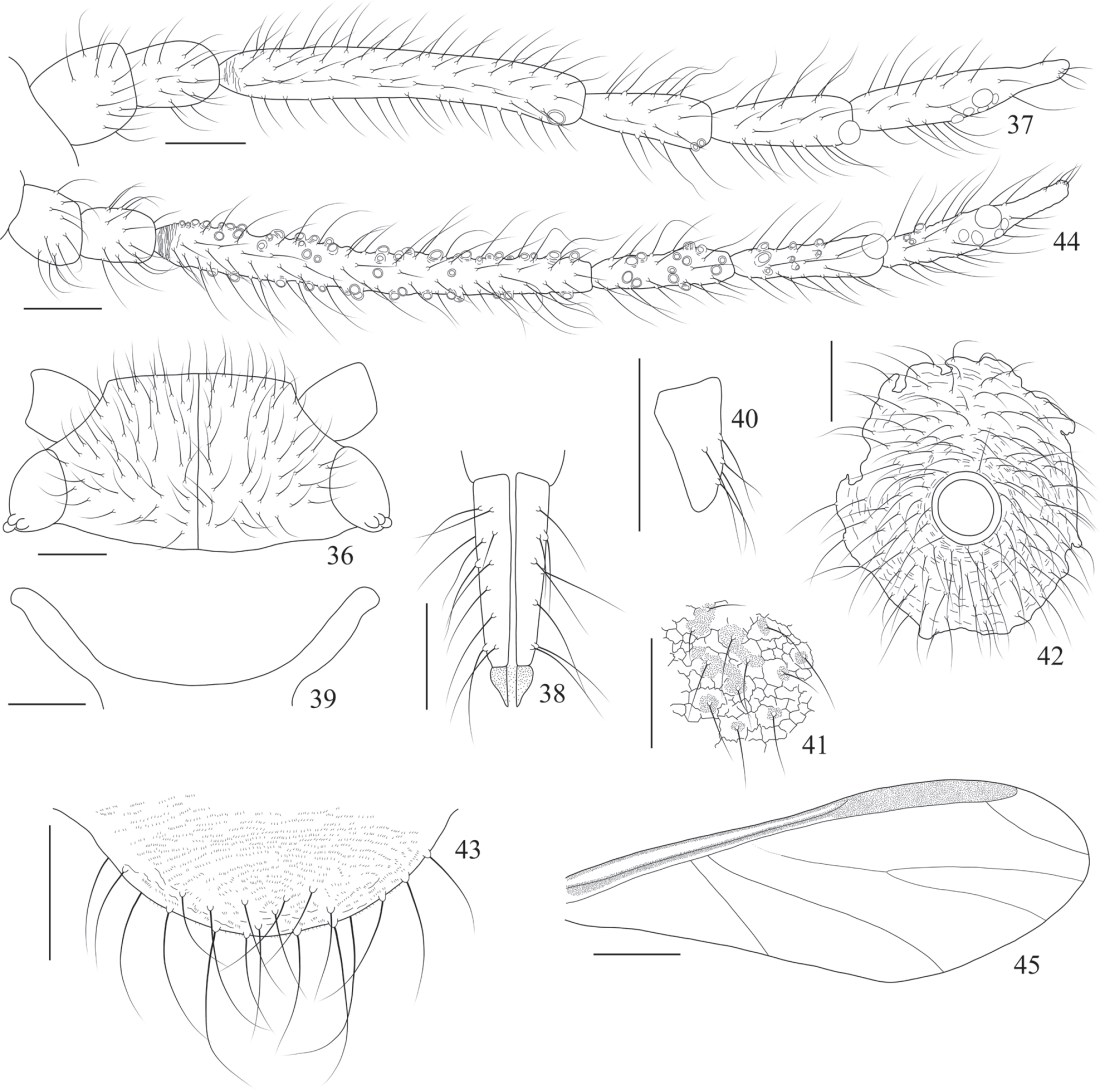
*Sinolachnusyunnanensis* Qiao & Li, sp. nov. Apterous viviparous female: **36** dorsal view of head **37** antenna **38**URS**39** mesosternal furca **40** hind first tarsal segment **41** reticulations and setae bearing dark base-sclerites on spinal part of abdominal tergites **42** siphunculus **43** cauda. Alate viviparous female: **44** antenna **45** fore wing. Scale bars: 0.10 mm (**36–44**); 0.50 mm (**45**). (Figs 36, 37 and 40 were drawn according to type material No. 54272, Figs 38 and 39 according to No. 54223, Fig. 41 according to No. 54224, Figs 42–44 according to No. 54211, Fig. 45 according to No. 54113)

***Thorax.*** Metanotum with small scattered sclerites on spino-pleural part, and 1 pair of marginal sclerites. Dorsal setae long and pointed. Mesosternal furca with a short stem (Figs 39, 50). Legs normal, with long and pointed setae. First tarsal chaetotaxy: 9–12, 8–10, 5–8; first fore tarsal segments with 3–7 peg-like setae and 3–7 long setae, first mid-tarsal segments with 2–4 peg-like setae and 5–8 long setae, first hind tarsal segments with 0–2 peg-like setae and 4–6 long setae.

***Abdomen.*** Abdominal tergites I–VI with reticulations, tergites VII, VIII and venter with spinulose stripes. Abdominal tergite I and marginal part of tergite II with small scattered sclerites; tergites II–V with scattered spinal sclerites, sometimes fused (Figs 41, 52) or unobvious; tergite VI often with scattered spino-pleural sclerites, sometimes fused; tergite VII with a broad transverse patch with irregular margin; tergite VIII with a narrow band, sometimes separated in the middle; intersegmental muscle sclerites small and dark. Dorsal setae fine, long and pointed. Abdominal tergite VIII with 36–65 setae. Spiracles oval, closed, on brown spiracular plates. Siphunculi truncate, on dark brown seta-bearing cones, with flange and transverse striae (Figs 42, 53), surrounding by 135–195 setae. Cauda round with spinulose stripes, with 33–45 long or short setae (Figs 43, 54). Anal plate broadly round with spinules, with 77–95 long or short setae (Fig. 55). Genital plate transverse elliptical, anterior part slightly concaved, with spinulose stripes, with 120–148 setae (Fig. 56). Genopophyses three, each with 6–7, 6–10, 6–7 setae.

**Figures 46–58.**
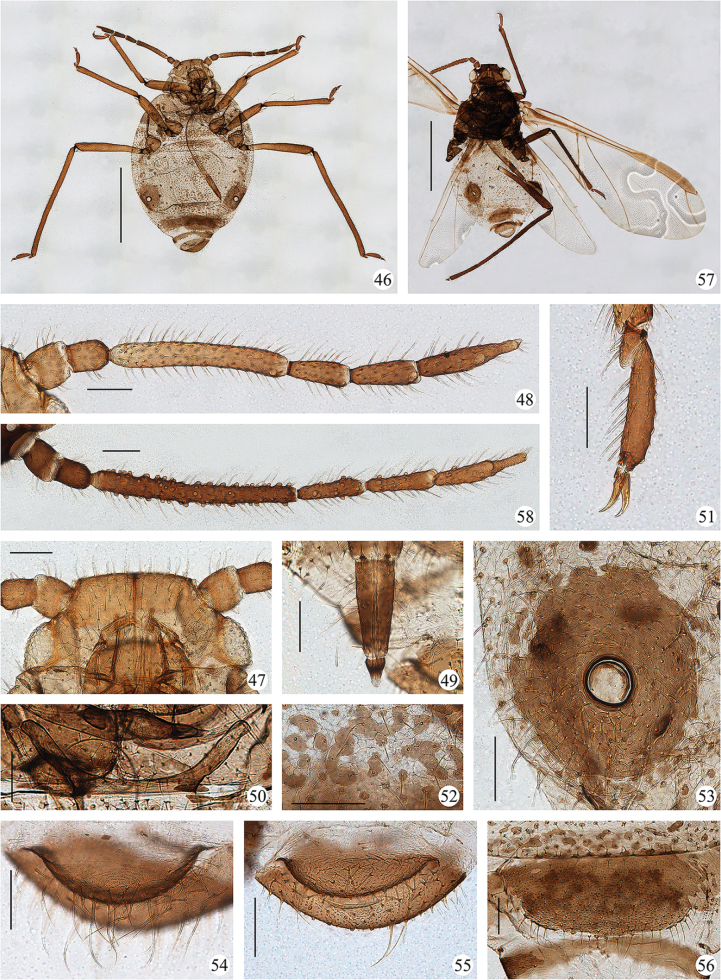
*Sinolachnusyunnanensis* Qiao & Li, sp. nov. Apterous viviparous female: **46** dorsal view of body with large sclerites **47** dorsal view of head **48** antenna **49**URS**50** mesosternal furca **51** hind tarsi and claws **52** reticulations and setae bearing dark base-sclerites on spinal part of abdominal tergites **53** siphunculus **54** cauda **55** anal plate **56** genital plate. Alate viviparous female: **57** dorsal view of body **58** antenna. Scale bars: 1.00 mm (**46, 57**); 0.10 mm (**47–56, 58**). (Fig. 46 was photographed according to type material No. 54424, Figs 47, 48, 51 and 52 according to No. 54272, Figs 49 and 50 according to No. 54223, Figs 53, 54, 56 and 58 according to No. 54211, Fig. 55 according to No. 54252, Fig. 57 according to No. 54113)

**Alate viviparous female**: Body elongate oval, head and thorax blackish brown, abdomen brown in life (Fig. 64); antennae, legs, siphunculi and patches on abdominal tergites VII and VIII blackish-brown.

**Figures 59–64.**
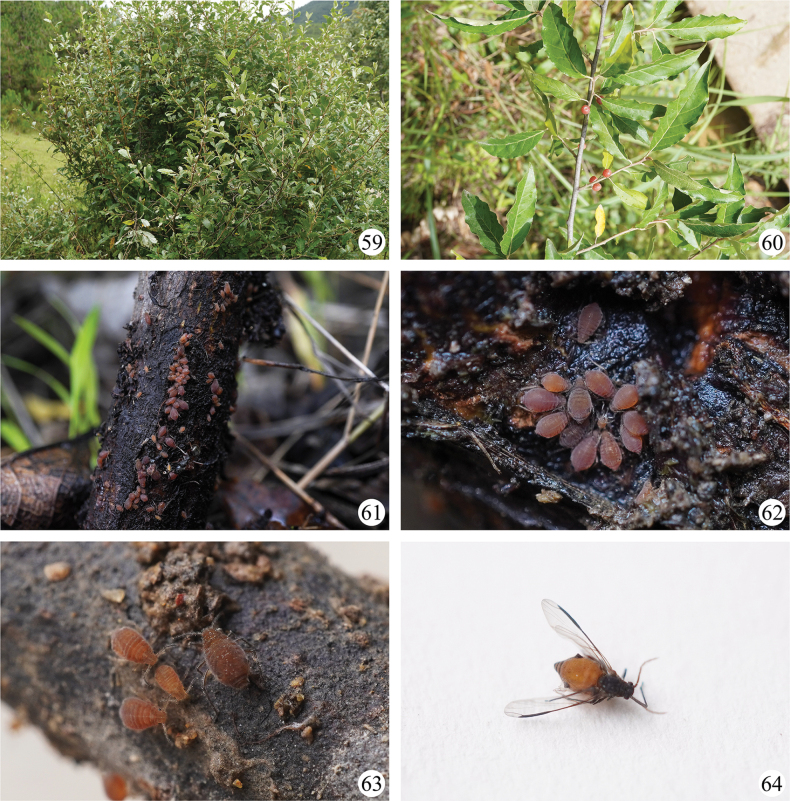
*Sinolachnusyunnanensis* Qiao & Li, sp. nov. **59, 60** host plant **61–63** apterous viviparous females and nymphs on stems of host plants near the ground **64** alate viviparous female.

***Mounted specimens.*** Head, antennae, rostral segments III–V, thorax, legs except basal part of femora, siphunculi, cauda, anal plate and genital plate dark brown, other parts pale brown; dorsal and ventral setae on abdomen bearing dark base-sclerites. For morphometric data, see Table 2.

***Head.*** Head dorsum smooth, with an obvious dark median suture. Head with 71–79 long and pointed dorsal setae. Frons flat. Ocular tubercles well developed. Antennae almost smooth (Figs 44, 58), basal part of Ant. III and PT with transverse striae. Antennal setae fine, most long and pointed, few short and blunt, Ant. I–VI each with 19–23, 30, 101, 33, 32, 32+7 setae, respectively; PT with 5 short blunt setae at apex. Primary rhinaria round, Ant. VI with 4 accessory rhinaria around primary rhinaria; secondary rhinaria round and protuberant, Ant. III–VI with 70–80, 14, 8, 3 secondary rhinaria, respectively. Rostrum long, reach abdominal segment IV; URS elongate wedge-shaped, with 3 pairs of primary setae and 18 accessory setae.

***Thorax.*** Legs normal, with long and pointed setae. First tarsal chaetotaxy: 7–10, 6–10, 4; first fore tarsal segments with 4 or 5 peg-like setae and 3–5 long setae, first mid-tarsal segments with 2–5 peg-like setae and 4 or 5 long setae, first hind tarsal segments with none or 1 peg-like setae and 3 or 4 long setae. Wings with scaly imbrications entirely (Fig. 57); campaniform sensilla near the base of subcosta slightly protuberant, fore wings and hind wings each with 10–14 and 7–9 campaniform sensilla on basal part, respectively; fore wings with pterostigma elongate, pale media once branched and faint on basal part (Fig. 45); hind wings with two oblique veins.

***Abdomen.*** Abdominal tergites smooth, reticulations obvious or not; tergites VII, VIII and venter with spinulose stripes. Abdominal tergites I–III each with 1 pair of marginal sclerites, tergites V and VI with a few scattered spinal sclerites, tergite VII with a broad transverse patch with irregular margin; tergite VIII with a narrow band; intersegmental muscle sclerites small and dark. Setae on abdominal tergites fine, most long and pointed, few short and blunt, dorsal setae sparser than on venter. Abdominal tergite VIII with 28 setae. Spiracles oval and closed, on brown spiracular plates. Siphunculi truncate, on dark brown seta-bearing cones, apical with few transverse striae and flange, surrounding by 140–156 setae. Cauda round with spinulose stripes, with 32–43 long or short setae. Anal plate broadly round with spinules, with 71–78 long or short setae. Genital plate transverse elliptical with spinulose stripes, with 110–124 setae. Genopophyses three, each with 7, 8, 7 setae.

#### Distribution.

China (Yunnan).

#### Host plant.

*Elaeagnus* sp. (Elaeagnaceae).

#### Biology.

The species colonizes branches and stems of host plants near the ground under ant nests.

#### Comments.

Apterae of the new species resemble *Sinolachnusrubi*, which is only known from apterous viviparous females, in having abdominal tergites with scattered spinal sclerites, sometimes fused, but differs from it as follows: body relatively small, 1.94–2.64 mm in length (the latter: 2.70–3.40 mm); Ant. VI 0.50–0.66 times as long as Ant. III (the latter: 0.40–0.42 times); secondary rhinaria often absent, Ant. III and IV with 1 or 2, Ant. VI with 1 occasionally (the latter: Ant. III–VI with 1–7, 1–6, 1–5, 1–3 secondary rhinaria, respectively); Setae on Ant. III 3.19–4.86 times as long as Ant. III BD (the latter: 2.12–2.75 times); URS with 18–26 secondary setae (the latter: URS with 11 or 12 secondary setae); Setae on Hind tibiae 1.48–2.14 times as long as MW hind tibia (the latter: 0.90–1.20 times); HT Ib 2.40–3.60 times as long as HT Id (the latter: 1.80–2.00 times); abdominal tergite VIII with 36–65 setae (the latter: 18–20 setae), Setae on Tergite VIII 3.61–5.33 times as long as Ant. III BD (the latter: 2.70–3.40 times).

Alatae of the new species resemble *S.nipponicus*, which is only known from alate viviparous females, in having the body relatively small (body length less than 3.00 mm), Ant. III with fewer secondary rhinaria (66–88 secondary rhinaria), PT with several long setae on basal part, media of fore wings once branched, but differs from it as follows: PT 0.57 times as long as Ant. VIb (the latter: 0.73–0.83 times); Ant. VIb with 32 setae (the latter: 21–23 setae); Setae on Hind tibiae 0.10–0.12 mm (the latter: 0.070–0.075 mm); abdominal tergites V and VI with few scattered spinal sclerites, tergite VII with a broad transverse patch with irregular margin (the latter: abdominal tergites I–VII without spinal and pleural patches); genital plate transverse elliptical (the latter: genital plate with irregular and divided proximal part).

### ﻿Keys to the species of *Sinolachnus* in China

Apterous viviparous females

**Table:** 

1	Body length 1.94–2.64 mm; antenna 0.93–1.39 mm in length; Ant. IV with 0–2 secondary rhinaria	**2**
–	Body length 2.77–3.67 mm; antenna 1.48–1.84 mm in length; Ant. IV with 2–11 secondary rhinaria	**3**
2	Ant. IV slightly longer than Ant. V, Ant. VI 0.44 times as long as Ant. III, Ant. V with 1 secondary rhinarium	***S.niitakayamensis* (Takahashi)**
–	Ant. IV slightly shorter than Ant. V, Ant. VI 0.50–0.66 times as long as Ant. III, Ant. V without secondary rhinaria	***S.yunnanensis* Qiao & Li, sp. nov.**
3	URS 0.65–0.81 times as long as HT II, with 10–14 accessory setae; first tarsal segments with 8–16 setae; abdominal tergites with sclerites	***S.rubusis* Qiao & Li, sp. nov.**
–	URS 0.92–1.02 times as long as HT II, with 20–24 accessory setae; first tarsal segments with 1–8 setae; abdominal tergites without sclerites	***S.yushanensis* Kanturski, Yeh & Lee**

Аlate viviparous females

**Table:** 

1	Media of fore wings once branched	**2**
–	Media of fore wings twice branched	**5**
2	Ant. III–V with 70–80, 14, 8 secondary rhinaria, respectively; Setae on Ant. III 3.93 times as long as Ant. III BD, basal part of PT with 5 long setae	***S.yunnanensis* Qiao & Li, sp. nov.**
–	Ant. III with more than 100, Ant. IV and V each with more than 20 secondary rhinaria; Setae on Ant. III 2.10–2.85 times as long as Ant. III BD, basal part of PT without long setae	**3**
3	Ant. III with 220–255 and Ant. IV with 50–70 secondary rhinaria; fore wings with scaly imbrications mostly on distal part	***S.takahashii* Kanturski, Yeh & Lee**
–	Secondary rhinaria on Ant. III more than 200, on Ant. IV less than 40; fore wings with scaly imbrications entirely	**4**
4	PT 0.57–0.62 times as long as Ant. VIb, Ant. VI with 4–6 secondary rhinaria; URS 0.79–0.82 times as long as HT II; hind wings with 9–11 pseudo-sensoria on basal part	***S.niitakayamensis* (Takahashi)**
–	PT 0.35–0.42 times as long as Ant. VIb, Ant. VI with 7–16 secondary rhinaria; URS 0.88–0.96 times as long as HT II; hind wings with 17–19 pseudo-sensoria on basal part	***S.yushanensis* Kanturski, Yeh & Lee**
5	Body length about 2 mm; antenna about 0.68 times as long as body length; fore wings with scaly imbrications mostly on distal part	***S.taiwanus* Tao**
–	Body length more than 3 mm; antenna 0.45–0.58 times as long as body length; fore wings with scaly imbrications entirely	**6**
6	Antenna 0.58 times as long as body length; Ant. III–VI with 280–285, 62–81, 64–89, 22–39 small secondary rhinaria, respectively; abdominal tergite VII with a sclerotic band	***S.plurisensoriatus* (Zhang)**
–	Antenna 0.45–0.51 times as long as body length; Ant. III–VI with 29–54, 5–18, 3–14, 4–8 secondary rhinaria, respectively, secondary rhinaria in various sizes; abdominal tergite VII without sclerites	***S.rubusis* Qiao & Li, sp. nov.**

## Supplementary Material

XML Treatment for Sinolachnus rubusis

XML Treatment for Sinolachnus yunnanensis

## ﻿Additional information

### Author ORCIDs

Zhao-Xu Li https://orcid.org/0000-0002-9252-4877

Jing Chen https://orcid.org/0000-0002-7584-5249

Li-Yun Jiang https://orcid.org/0000-0002-2527-9613

Ge-Xia Qiao https://orcid.org/0000-0002-7300-6812

### Data availability

All of the data that support the findings of this study are available in the main text.
